# Association of maternal birth weight with the risk of low birth weight and small-for-gestational-age in offspring: A prospective single-center cohort study

**DOI:** 10.1371/journal.pone.0251734

**Published:** 2021-05-14

**Authors:** Megumi Shibata, Kohei Ogawa, Seiji Kanazawa, Maki Kawasaki, Naho Morisaki, Asako Mito, Haruhiko Sago, Reiko Horikawa, Naoko Arata

**Affiliations:** 1 Center for Maternal-Fetal, Neonatal and Reproductive Medicine, National Center for Child Health and Development, Tokyo, Japan; 2 Department of Social Medicine, National Research Institute for Child Health and Development, Tokyo, Japan; 3 Division of Endocrinology and Metabolism, National Center for Child Health and Development, Tokyo, Japan; Kobe University Graduate School of Medicine School of Medicine, JAPAN

## Abstract

**Background:**

Although low birth weight in Japan has slightly increased over the past several decades, the association between maternal birth weight and pregnancy outcomes remains poorly understood.

**Methods:**

In this hospital-based, prospective cohort study conducted at the National Center for Child Health and Development, we obtained information on pregnant women’s birth weight via their maternal and child health handbook. We analyzed 944 women born at term after dividing them into five categories according to their birth weight: <2500 g, 2500–2999 g, 3000–3499 g, 3500–3999 g, and ≥4000 g. Multivariate logistic regression analysis and trend analysis were used to elucidate the extent to which maternal birth weight was associated with small-for-gestational-age and low birth weight in offspring, as well as with hypertensive disorders of pregnancy.

**Results:**

Compared with women with a birth weight of 3000–3499 g, those born with a birth weight <2500 g had a significantly higher risk of low birth weight (adjusted odds ratio: 5.39, 95% confidence interval: 2.06–14.1) and small-for-gestational-age (adjusted odds ratio: 9.11, 95% confidence interval: 3.14–26.4) infants. No significant association was found between the incidence of hypertensive disorders of pregnancy and preterm birth. A linear relationship was observed between the lower birth weight categories and a higher risk of low birth weight and small-for-gestational-age (p-values for trends: 0.009 and <0.001, respectively), but no linear relationship was observed for the risk of preterm birth and hypertensive disorders of pregnancy (p-value for trends: 0.317 and 0.157, respectively).

**Conclusions:**

Our findings suggest that lower maternal birth weight is associated with small-for-gestational-age and low birth weight in offspring of women born at term.

## Introduction

The risk of the development of chronic diseases, such as diabetes mellitus, hypertension, and obesity, in adulthood is influenced by environmental factors in early life, including the fetal period [1, 2]. Several recent studies have found a possible inverse association between maternal birth weight and the risk of adverse birth outcomes, such as preterm birth, low birth weight (LBW), and small-for-gestational-age (SGA) [3–6], in offspring, as well as maternal hypertensive disorders of pregnancy (HDP) [7–10]. To date, most studies on the association between maternal birth weight and pregnancy outcomes have enrolled Caucasian subjects, and no study enrolling Asian subjects has been conducted. However, the rate of LBW is higher in Asia; [11] in particular, the average birth weight among Japanese women has decreased by approximately 200 g from that in the 1980s [12, 13]. Thus, understanding whether there is an intergenerational effect due to Japanese women being born with a lower birth weight on increasing the risk of underweight offspring is of great importance in assessing the healthcare needs of future generations.

Lower birth weight comprises both preterm births and SGA. Preterm births in Japan have slightly increased over the past several decades [14], and some studies suggest that inadequate prepregnant body mass index (BMI) and weight gain during pregnancy is a crucial cause of lower birth weight [15, 16]. Thus, to determine whether and to what extent women born at term with SGA had a higher risk of adverse birth outcomes in their own offspring, we investigated the intergenerational effect of birth weight among Japanese women born at term by examining the effect of maternal birth weight on SGA and LBW in their offspring, as well as HDP.

## Materials and methods

### Study design and subjects

We obtained data for this prospective cohort study conducted at National Center for Child Health and Development (NCCHD) in Japan from maternal and child health handbooks. Pregnant women were recruited at their antenatal visit in the first or second trimester between April 2010 and November 2013 and were asked to bring their maternal and child health handbook to subsequent prenatal visits. The maternal and child health handbook is a very popular, prospective form of record-keeping in Japan designed to record specific test results, such as those of urinalysis; any events occurring during the perinatal period; and other data points, including blood pressure, neonatal birth weight, and the gestational week at birth. The data in the handbook are recorded by healthcare providers.

Women with multiple pregnancies, missing data on birth weight in their maternal and child health handbook, or a history of childbirth at another hospital besides the NCCHD were excluded. Women who were born preterm were also excluded to eliminate the possible effect of maternal preterm birth on pregnancy outcomes. Furthermore, women with missing data on maternal demographics were excluded. All participants gave their written informed consent at the time of recruitment. The study protocol was approved by the Institutional Review Board at the NCCHD in May 2011 (No. 484) and was performed in accordance with the guidelines of the Declaration of Helsinki and other nationally valid regulations.

### Variables of interest

The primary variable of interest was the birth weight of pregnant women, as documented in their maternal and child health handbook. Pregnant women were categorized by their own birth weight into the following groups: <2500 g, 2500–2999 g, 3000–3499 g, 3500–3999 g, and ≥4000 g.

We considered the adverse birth outcomes of preterm birth, LBW, and SGA, all of which were calculated from the gestational age (days) and birth weight (g) in the medical records, as well as the presence of HDP. We defined preterm birth as <37 weeks of gestation [17, 18], LBW as <2500 g, and SGA as birth weight below the 10th percentile for gestational age on the birth weight reference chart [19]. HDP was defined as an antenatal systolic blood pressure >140 mmHg or a diastolic blood pressure >90 mmHg, regardless of gestational age and the presence of proteinuria [20].

Other variables pertaining to the mother and grandmother were categorized as follows: age at delivery (aged ≤30, 30–34, or ≥35 years), parity (primipara or multipara), and prepregnancy BMI (≤18.5, 18.5–25, or ≥25 kg/m^2^). Information on covariates and adverse outcomes was also retrieved from the medical records.

### Statistical analysis

First, we compared the baseline characteristics according to the subjects’ birth weight using one-way ANOVA for continuous variables and the chi-squared test for dichotomous variables. Second, we conducted linear tests for trends between the birth weight categories and the risk of outcomes and used a multivariate logistic regression analysis to estimate the effect of the subjects’ birth weight on the risk of adverse birth outcomes, using women born with a birth weight of 3000–3499 g as the reference. In line with previous studies, we considered three demographic factors in pregnant women, namely, age, parity, and prepregnancy BMI [3–5, 7]. Because the demographic data of many of the grandmothers were missing, we additionally performed a sensitivity analysis that further adjusted two of the grandmothers’ demographics, namely, age and parity [4, 5].

All statistical analyses were conducted using Stata v14 software (StataCorp, College Station, TX). P-values <0.05 were considered statistically significant.

## Results

Of the 1,028 women participating in the study, one with a current multiple pregnancy, 17 with missing data on their own birth weight or weeks of gestation, 25 who were born preterm, and 41 with missing data on their prepregnancy BMI were excluded, leaving 944 women for enrollment in this study. The characteristics of the 944 pregnant women and their mothers are presented in Table 1. Women with a higher birth weight had a mother with a higher average age at pregnancy (p = 0.004), higher average prepregnancy BMI (p = 0.001), and lower primiparity rate (p < 0.001). On the other hand, there was no difference in the average age at pregnancy, rate of primiparity, and average prepregnancy BMI for current pregnancies between the subjects’ birth weight categories. As for neonatal information at birth, women with higher birth weight were likely to have a child with a heavier birth weight (p < 0.001) and greater height (p < 0.001).

**Table 1:** Characteristics of pregnant women’s mothers and pregnant women (N = 944) by categories of women’s birth weight.

Demographic factor	Categories of women’s birth weight					
	<2500 g (n = 23)	2500–2999 g (n = 280)	3000–3499 g (n = 444)	3500–3999 g (n = 168)	>4000 g (n = 29)	P-value^a^
**Demographics for pregnant women’s mother**						
**Age, years**	25.6 (2.6)	27.3 (3.3)	27.3 (3.3)	27.9 (3.1)	29.4 (3.4)	0.004
**Missing (n = 342)**	*9*	*107*	*151*	*69*	*6*	
**Primiparous (%)**	13 (68.4)	109 (54.5)	160 (49.7)	34 (29.6)	8 (38.1)	<0.001
**Missing (n = 267)**	*4*	*80*	*122*	*53*	*8*	
**Prepregnancy BMI, kg/m**^**2**^	19.5 (2.6)	19.4 (1.9)	20.1 (1.8)	20.4 (1.9)	21.5 (1.9)	0.001
**Missing (n = 648)**	*17*	*190*	*301*	*122*	*18*	
**Demographics for pregnant women**						
**Age, years**	34.7 (4.8)	35.4 (4.2)	35.4 (4.1)	36.4 (4.4)	35.4 (3.6)	0.096
**Primiparous (%)**	19 (82.6)	175 (62.5)	280 (63.1)	111 (66.1)	17 (58.6)	0.338
**Prepregnancy BMI, kg/m**^**2**^	20.6 (2.2)	20.3 (2.7)	20.3 (2.4)	20.5 (2.5)	21.7 (3.4)	0.062
**Neonatal information at birth**						
**Gestational week at delivery**	38.6 (1.6)	38.5 (1.8)	38.8 (1.5)	38.9 (1.6)	39.0 (1.7)	0.094
**Birth weight, g**	2798 (510)	2870 (421)	3043 (424)	3104 (459)	3253 (482)	<0.001
**Birth height, cm**	47.9 (3.3)	48.7 (2.7)	49.4 (2.2)	49.6 (2.4)	49.8 (2.0)	<0.001

BMI: body mass index.

Values are presented as mean (SD) or number (percentage)

Missing data are indicated in italics.

^a^One-way ANOVA analysis for continuous variables and chi-squared test for dichotomous variables.

The association between women’s birth weight and their pregnancy outcomes is shown in Table 2. Compared with women born with a birth weight of 3000–3499 g, those born at <2500 g had a higher risk of LBW (adjusted odds ratio [OR]: 95% confidence interval [CI): 5.39 (2.06–14.1) and SGA (OR: 9.11, 95% CI: 3.14–26.4) offspring. Furthermore, we observed an inverse linear trend in the subjects’ birth weight on the risk of LBW (p for trend = 0.009) and SGA (p for trend <0.001). In contrast, we failed to detect a linear association of the subjects’ birth weight with the risk of preterm birth and HDP (p for trend = 0.317 and 0.157, respectively).

**Table 2:** Main analysis (N = 944) among women born at term.

Birth weight of pregnant women themselves	Preterm birth			Low birth weight			SGA			HDP		
	Prevalence (%)	OR (95% CI)	aOR^a^ (95% CI)	Prevalence (%)	OR (95% CI)	aOR^a^ (95% CI)	Prevalence (%)	OR (95% CI)	aOR^a^ (95% CI)	Prevalence (%)	OR (95% CI)	aOR^a^ (95% CI)
<2500 g	2/23 (8.7)	1.67 (0.37–7.53)	1.79 (0.39–8.17)	7/23 (30.4)	4.81 (1.86–12.4)	5.39 (2.06–14.1)	6/23 (26.1)	8.87 (3.10–25.3)	9.11 (3.14–26.4)	2/23 (8.7)	3.16 (0.67–14.9)	2.65 (0.55–12.7)
2500–2999 g	23/280 (8.2)	1.57 (0.87–2.83)	1.56 (0.86–2.82)	37/280 (13.2)	1.67 (1.03–2.71)	1.68 (1.03–2.72)	33/280 (11.8)	3.36 (1.83–6.15)	3.35 (1.82–6.15)	12/280 (4.3)	1.48 (0.67–3.30)	1.45 (0.65–3.26)
3000–3499 g	24/444 (5.4)	reference	reference	37/444 (8.3)	reference	reference	17/444 (3.8)	reference	reference	13/444 (2.9)	reference	reference
3500–3999 g	9/168 (5.4)	0.99 (0.45–2.18)	0.99 (0.45–2.18)	16/168 (9.5)	1.16 (0.63–2.71)	1.19 (0.64–2.20)	9/168 (5.4)	1.42 (0.62–3.25)	1.52 (0.66–3.49)	4/168 (2.4)	0.81 (0.26–2.52)	0.78 (0.25–2.46)
>4000 g	3/29 (10.3)	2.02 (0.57–7.15)	1.73 (0.48–6.26)	2/29 (6.9)	0.81 (0.19–3.56)	0.85 (0.19–3.73)	1/29 (3.5)	0.90 (0.12–6.99)	0.95 (0.12–7.43)	1/29 (3.5)	1.18 (0.15–0.94)	0.99 (0.12–8.03)
		p for trend 0.375	p for trend 0.317		p for trend 0.009	p for trend 0.009		p for trend <0.001	p for trend <0.001		p for trend 0.151	p for trend 0.157

SGA: small-for-gestational-age, HDP: hypertensive disorders of pregnancy, OR: odds ratio, aOR: adjusted odds ratio, CI: confidence interval.

^a^Adjusted by maternal age at delivery, parity, and prepregnancy body mass index.

The results of a sensitivity analysis adjusted by grandmothers’ age and parity are shown in S1 Table. The odds ratios and the p-values for trends observed in the sensitivity analysis were similar to those observed in the primary analysis.

## Discussion

In line with previous studies, we observed that among women born at term, maternal birth weight had a significant inverse linear trend in terms of the risk of having offspring with LBW and SGA. In contrast, we failed to find any association between maternal birth weight and the risk of preterm birth and developing HDP. This is the first study to examine birth weight and the risk of adverse pregnancy outcomes in Japanese women.

We found a significant inverse relationship between maternal birth weight and the risk of LBW and SGA in the subjects’ offspring among women born at term. Although low birth weight has two causes, namely, SGA and preterm birth, our findings suggested a transgenerational vicious cycle in which lower maternal birth weight was an independent risk factor for lower birth weight in offspring. This also implied that SGA caused by reasons other than the transgenerational effect may lead to the onset of a new vicious cycle. Thus, it is crucial to avoid new occurrences of this vicious cycle caused by other controllable factors. Changing dietary patterns and body consciousness in reproductive-aged Japanese women and the relatively strict recommended limit on weight gain during pregnancy have been proposed as the major reasons for the decreasing trend in the birth weight of Japanese infants [15, 16, 21]. A possible remedy for this vicious cycle may be to educate reproductive-aged Japanese women about weight control before and during their pregnancy. Informing women about the possible adverse effects of LBW may motivate them to maintain adequate weight during pregnancy by possibly generating a desire to avoid adverse effects on their offspring because a study has suggested that perceived ideal weight gain influences actual weight gain [21].

We failed to find a significant association between maternal birth weight and the risk of preterm delivery. Previous studies investigating this association have shown inconsistent results. Two studies in Brazil and the USA that enrolled 1,982 and 2,678 women, respectively, showed that lower maternal birth weight (including preterm births) was significantly associated with a higher risk of preterm offspring [3, 6]. However, this effect may be due, at least in part, to the increased risk of preterm birth among women who were born preterm because previous studies have shown a vicious cycle in preterm births across generations [22–25]. In contrast, a US study enrolling 1,348 women reported a non-significant association [26], in line with our own. Although our study on women born at term indicated that lower birth weight was not an independent risk factor for preterm delivery, the sample size was limited. Thus, further research with a larger population is warranted to eliminate the effect of the subjects’ own history of preterm birth.

We failed to find a significant association between women born with lower birth weight and those who experience HDP, inconsistent with previous studies showing increased HDP risk among women with LBW [7–9]. A possible explanation for this discrepancy may be due to the small number of women with HDP compared with that in previous studies (67–2180 women with HDP were included), leading to a lack of statistical power. Because HDP is one of the most important morbidities related to SGA and LBW, further studies on a larger population should be undertaken to study whether HDP accounts for the association between women born with lower birth weight and risk of having offspring with LBW and SGA.

Although comparable in size with previous studies, the present study has the advantage of more accurate data on birth weight due to recording of the information by healthcare providers using the maternal and child health handbook, thereby minimizing recall bias [3, 26, 27]. Another advantage was the exclusion of women who were born preterm because they have a reportedly increased risk of preterm offspring. Nonetheless, our study has several limitations. First, our database did not include the socioeconomic status of the subjects, such as smoking status, household income, and the education level of the grandmother, although these factors might be associated both with maternal birth weight and pregnancy outcomes, including preterm birth and LBW [28, 29]. Second, although we conducted multivariate analysis adjusted by the grandmother’s age and parity, considerable data on the grandmothers’ characteristics were missing. Thus, further analysis, based on subjects where only a small amount of data pertaining to the grandmothers’ demographics are missing, is necessary.

## Conclusions

Our results suggest that birth weight is inversely related to the risk of SGA and LBW in offspring among Japanese women born at term and contributes to a vicious cycle in the intergenerational relationship of adverse birth outcomes. These findings may pave the way for interventions to prevent LBW and SGA, not least by motivating young Japanese women to better manage weight gain during pregnancy.

## Supporting information

S1 Table Sensitivity analysis adjusted by age and parity of women’s mothers at delivery (n = 486). (DOCX)
