# Supplementary material for: Association of maternal birth weight with the risk of low birth weight and small-for-gestational-age in offspring: A prospective single-center cohort study
Source: PLoS One. 2021 May 14;16(5):e0251734. doi: 10.1371/journal.pone.0251734 (PMC8121327; doi:10.1371/journal.pone.0251734)
Supplement: S1 Table — (DOCX) [file pone.0251734.s001.docx]

**S1 Table. Sensitivity analysis adjusted by age and parity of women’s mothers at delivery (n = 486).**

| **Birth weight of pregnant women themselves** | **Preterm birth** | | **Low birth weight** | | **SGA** | | **HDP** | |
| --- | --- | --- | --- | --- | --- | --- | --- | --- |
|  | **Prevalence** | **aOR (95% CI)** | **Prevalence** | **aOR (95% CI)** | **Prevalence** | **aOR (95% CI)** | **Prevalence** | **aOR (95% CI)** |
| **<2500 g** | 1/12 (8.3) | 1.64 (0.19–14.3) | 6/12 (50.0) | 10.9 (3.11–37.9) | 5/12 (41.7) | 15.9 (3.95–64.4) | 2/12 (16.7) | 3.03 (0.54–16.9) |
| **2500–2999 g** | 13/135 (9.6) | 2.03 (0.89–4.61) | 18/135 (13.3) | 1.48 (0.77–2.87) | 16/135 (11.9) | 2.77 (1.23–6.24) | 6/135 (4.4) | 0.91 (0.31–2.65) |
| **3000–3499 g** | 12/242 (5.0) | reference | 23/242 (9.5) | reference | 11/242 (4.6) | reference | 10/242 (4.1) | reference |
| **3500–3999 g** | 4/78 (5.1) | 1.05 (0.32–3.40) | 9/78 (11.5) | 1.26 (0.55–2.89) | 6/78 (7.7) | 2.00 (0.70–5.75) | 2/78 (2.6) | 0.59 (0.12–2.85) |
| **>4000 g** | 2/19 (10.5) | 2.03 (0.40–10.2) | 2/19 (10.5) | 1.20 (0.25–5.64) | 0/19 (0.0) | – | 1/19 (5.3) | 1.09 (0.12–9.68) |
| **–** | – | p for trend = 0.362 | – | p for trend = 0.047 | – | p for trend = 0.002 | – | p for trend = 0.428 |

SGA: Small-for-gestational-age, HDP: hypertensive disorders of pregnancy, aOR: adjusted odds ratio, CI: confidence interval.
